# Host–Fungus Interactions Link Autoimmune and Immune-Mediated Inflammatory Diseases to Gastrointestinal Cancer: Lessons from KA/KA and *Card9^−/−^* Mouse Models

**DOI:** 10.3390/biology15201878

**Published:** 2026-10-18

**Authors:** Joyce Zhu, Diya Rawal, Adeline Cheng, Debra Tross, Xin Li, Teizo Yoshimura, Keqiang Chen, Yinling Hu, Feng Zhu

**Affiliations:** Cancer Innovation Laboratory, Center for Cancer Research, National Cancer Institute, National Institutes of Health, Frederick, MD 21702, USA

**Keywords:** host–fungus interactions, autoimmune disease, immune-mediated inflammatory disease, APECED, inflammatory bowel disease, IKKα, CARD9, gastrointestinal cancer, esophageal squamous cell carcinoma, colorectal cancer

## Abstract

Autoimmune diseases, immune-mediated inflammatory diseases, and cancer are distinct pathological conditions that can intersect through immune dysregulation, chronic inflammation, impaired host defense, and altered host–microbe interactions. In selected settings, autoimmune and immune-mediated inflammatory diseases are associated with increased gastrointestinal cancer risk. Although bacterial and viral contributions to inflammation-associated cancer have been extensively studied, the role of fungi remains less investigated. This Opinion article discusses host–fungus interactions through two experimental mouse models that connect altered immune tolerance, innate antifungal responses, and gastrointestinal tumorigenesis. Kinase-dead IKKα K44A knock-in mice develop defective central immune tolerance, APECED-like autoimmune manifestations, mucosal fungal infection, epithelial EGFR signaling, and esophageal squamous cell carcinoma. By contrast, *Card9^−/−^* mice provide a distinct model in which impaired innate antifungal immunity alters intestinal fungal ecology and myeloid-cell responses in colitis-associated colorectal cancer. In the CARD9 setting, fungi may exert pathogenic or protective effects depending on host genotype, immune context, fungal species, and mycobiome structure. Together, these models provide useful tools for investigating how fungi may contribute to gastrointestinal tumorigenesis through cancer-promoting inflammation and immune suppression in a context-dependent manner. However, these findings do not yet validate clinical strategies and should not be interpreted as broadly representative of all autoimmune or immune-mediated inflammatory diseases. Further mechanistic and human studies are needed.

## Simple Summary

Autoimmune and immune-mediated inflammatory diseases are characterized by alterations in immune tolerance, innate immune responses, mucosal barrier function, and host defense, which may increase susceptibility to fungal infection or fungal dysbiosis. This Opinion article focuses on two mouse models, KA/KA and *Card9^−/−^* mice, that illustrate distinct ways in which impaired immune regulation may interact with fungi during gastrointestinal tumorigenesis. These models suggest that fungi may have pathogenic or protective effects depending on host genotype, immune context, fungal species, and fungal-recognition pathways. Thus, KA/KA and *Card9^−/−^* mice provide useful tools for investigating the context-dependent role of fungi in gastrointestinal tumorigenesis. However, these findings do not yet validate clinical strategies and should not be interpreted as broadly representative of all autoimmune or immune-mediated inflammatory diseases. Further mechanistic and human studies are needed.

## 1. Introduction

The immune system protects the host from infection and malignancy through coordinated innate and adaptive immune responses while maintaining self-tolerance and tissue homeostasis [1,2]. Disruption of this balance can lead to excessive immune activation, loss of immune tolerance, and persistent tissue inflammation, contributing to autoimmune or immune-mediated inflammatory diseases, increased susceptibility to infection, and microbial dysbiosis, including fungal dysbiosis [3]. Conversely, impaired antitumor immunity, often associated with an immunosuppressive tumor microenvironment containing myeloid-derived suppressor cells (MDSCs), tumor-associated macrophages (TAMs), and exhausted T cells with elevated immune checkpoint receptor expression, enables cancer cells to evade immune surveillance and promotes tumor development [4].

Because autoimmune and immune-mediated inflammatory diseases and cancer differ substantially in immune regulation, their therapeutic strategies often differ as well [5]. Patients with autoimmune disease are commonly treated by suppressing excessive inflammation, whereas cancer therapy frequently aims to enhance antitumor immunity. These immune-modulating approaches can disturb immune homeostasis, promote autoimmune inflammation, alter the microbiome, or increase susceptibility to infections [6]. For example, patients with autoimmune diseases treated with TNF-α inhibitors may develop fungal infections, including candidiasis and aspergillosis [7], possibly due to impaired antifungal immunity and microbiome dysbiosis. In addition, patients with certain autoimmune or immune-mediated inflammatory diseases, including rheumatoid arthritis (RA), systemic lupus erythematosus (SLE), Sjögren’s syndrome (SS), autoimmune polyendocrinopathy-candidiasis-ectodermal dystrophy (APECED), and inflammatory bowel disease (IBD), may exhibit increased susceptibility to fungal infections and fungal dysbiosis [8,9,10,11,12].

Interestingly, cancer risk is increased in several patient populations with autoimmune or immune-mediated inflammatory diseases. For example, RA, SLE, SS, APECED, and IBD are associated with an elevated risk of malignancy relative to the general population. Reported malignancies include hematologic cancers, such as lymphoma, leukemia, and myeloma, as well as gastrointestinal (GI) cancers, including esophageal squamous cell carcinoma (ESCC) and colorectal cancer (CRC) [13,14,15,16,17,18,19,20,21,22]. These clinical observations raise the possibility that fungal infection or fungal dysbiosis may interact with chronic inflammation, impaired host defense, or altered mucosal immunity to influence cancer risk in selected disease contexts. Conversely, patients with cancer may develop autoimmune manifestations, particularly after immune checkpoint inhibitor therapy, which enhances antitumor immunity but can also disrupt immune tolerance and trigger immune-related adverse events (irAEs) [23,24,25,26,27].

Several mechanisms have been implicated in the association between autoimmunity and cancer, including chronic inflammation, impaired immune surveillance, treatment-related side effects, and impaired clearance of bacterial, viral, and fungal pathogens, as well as microbe dysbiosis [27,28,29,30]. Fungi are responsible for millions of infections and deaths worldwide each year, posing a significant threat to public health. In 2022, the World Health Organization listed 19 fungal priority pathogens, including *Aspergillus fumigatus*, *Candida albicans*, *Candida auris*, and *Cryptococcus neoformans* [31]. In addition to fungal infection, fungal dysbiosis has been associated with aberrant inflammatory responses in autoimmune-related inflammatory diseases such as IBD [12]. While previous studies have focused primarily on host–bacterial and host–viral interactions in cancer development, fewer studies have examined how host–fungus interactions may increase cancer risk in patients with autoimmune and immune-mediated inflammatory diseases [32]. Furthermore, the lack of appropriate autoimmune cancer animal models has limited mechanistic understanding of the relationship between fungal infections, immune dysregulation, and cancer development.

Host pattern recognition receptors (PRRs) recognize pathogen-associated molecular patterns (PAMPs) from fungal cell walls [33]. Among these receptors, C-type lectin receptors (CLRs) such as Dectin-1 and Dectin-2 play important roles in fungal recognition and signal through SYK and the adaptor protein caspase recruitment domain-containing protein 9 (CARD9), leading to activation of downstream inflammatory pathways, including NF-κB signaling [34]. IKKα is a key component of NF-κB signaling and is required for immune regulation, central tolerance, and host defense [35,36,37,38]. Notably, both CARD9 and IKKα have been associated with autoimmunity or immune-mediated inflammation, fungal infection or fungal dysbiosis, and GI cancers in humans and mouse models [37,38,39,40,41,42,43,44]. Two mouse models, kinase-dead IKKα K44A knock-in mice (KA/KA mice) and *Card9^−/−^* mice, provide valuable tools for investigating how impaired immune regulation and fungal infection or dysbiosis may contribute to GI tumorigenesis in a context-dependent manner [40,43,44].

This article is intended as an Opinion and model-focused review rather than a comprehensive systematic review of all evidence linking fungi, immune dysregulation, and cancer. We focus on two mouse models, KA/KA mice and *Card9^−/−^* mice, because they illustrate two distinct routes by which altered immune regulation may interact with fungal infection or fungal dysbiosis during GI tumorigenesis. The KA/KA model highlights defective central tolerance, autoreactive T-cell-mediated mucosal inflammation, fungal infection, epithelial EGFR signaling, and ESCC. By contrast, the *Card9^−/−^* model highlights impaired innate antifungal immunity, intestinal fungal dysbiosis or altered fungal sensing, altered myeloid-cell responses, impaired inflammasome activation, the IL-18–IFN-γ responses, and colitis-associated CRC. These models provide useful tools for investigating the context-dependent role of fungi in GI tumorigenesis. However, the findings from these models should not be interpreted as broadly representative of all autoimmune or immune-mediated inflammatory diseases. Further mechanistic and human studies are needed.

## 2. KA/KA Mice

IKKα is encoded by the *CHUK* gene and plays an important role in both canonical and non-canonical NF-κB pathways. In the canonical NF-κB pathway, IKKα forms the IKK complex together with IKKβ and IKKγ/NEMO, which phosphorylates IκB proteins and promotes their ubiquitination and subsequent proteasomal degradation, thereby allowing NF-κB activation [35,36]. In the non-canonical NF-κB pathway, IKKα homodimers phosphorylate p100 and promote its processing into p52, leading to activation of RelB/p52-dependent gene transcription [35]. Through NF-κB pathways, IKKα contributes to immune regulation, inflammation, host defense, and cancer-related biological processes [40]. IKKα deficiency or reduced expression has been associated with immunodeficiency, immune dysregulation, autoimmunity, recurrent infections, and cancer [37,40,45].

Recent studies have shown that KA/KA mice recapitulate several key features of APECED, including defective central tolerance, mucosal fungal infection, autoimmune manifestations, and ESCC [39,40]. APECED is a rare multiorgan autoimmune disorder caused by mutations in *AIRE*, which disrupt AIRE-dependent central immune tolerance established in the thymus and permit the escape of autoreactive T cells [11]. Patients with APECED develop chronic mucosal candidiasis in the oral cavity and esophagus as early as 5 years of age, and a subset of patients develop ESCC [17,18,19]. Although *Aire^−/−^* mice show autoimmune manifestations and increased susceptibility to *Candida albicans*, they do not develop spontaneous ESCC [46], limiting their usefulness for studying autoimmune-associated esophageal tumorigenesis.

Similar to patients with APECED, KA/KA mice develop fungal infections in the oral cavity and esophagus [40]. Approximately 20% of KA/KA mice also develop spontaneous ESCC. Internal transcribed spacer sequencing of oral swabs and esophageal samples identified 19 fungal species in KA/KA mice [40]. Among them, *Cladosporium cladosporioides* (*C. cladosporioides*) was the most abundant fungus in the oral cavity and the second most abundant in the esophagus [40]. *C. cladosporioides* is a common indoor and outdoor mold and has been associated with human head and neck SCC (HNSCC) [47,48,49]. Notably, repeated oral inoculation with 2 × 10^7^ live *C. cladosporioides* increased ESCC incidence in KA/KA mice to approximately 80%, whereas antifungal treatment reduced fungal burden and prevented ESCC development [40].

It has been reported that fungal colonization activates EGFR through fungus and epithelial cell interaction [50]. In addition, EGFR is a target of IKKα [51]. Interestingly, blockade of EGFR reduced fungal colonization in the esophagus of KA/KA mice, suggesting that EGFR activation increases fungal burden and that fungal infection may promote esophageal tumorigenesis, at least in part, through activation of epithelial EGFR signaling [40].

Further mechanistic studies showed that depletion of autoreactive T cells diminished fungal infection, macrophage infiltration, and ESCC formation in KA/KA mice [40]. These findings suggest that autoreactive T cell-mediated tissue injury and inflammation may create a permissive niche for fungal colonization and tumor-promoting inflammation. Supporting this idea, aspirin treatment or macrophage depletion with clodronate-loaded liposomes significantly reduced fungal burden in KA/KA mice.

**Figure 1:**
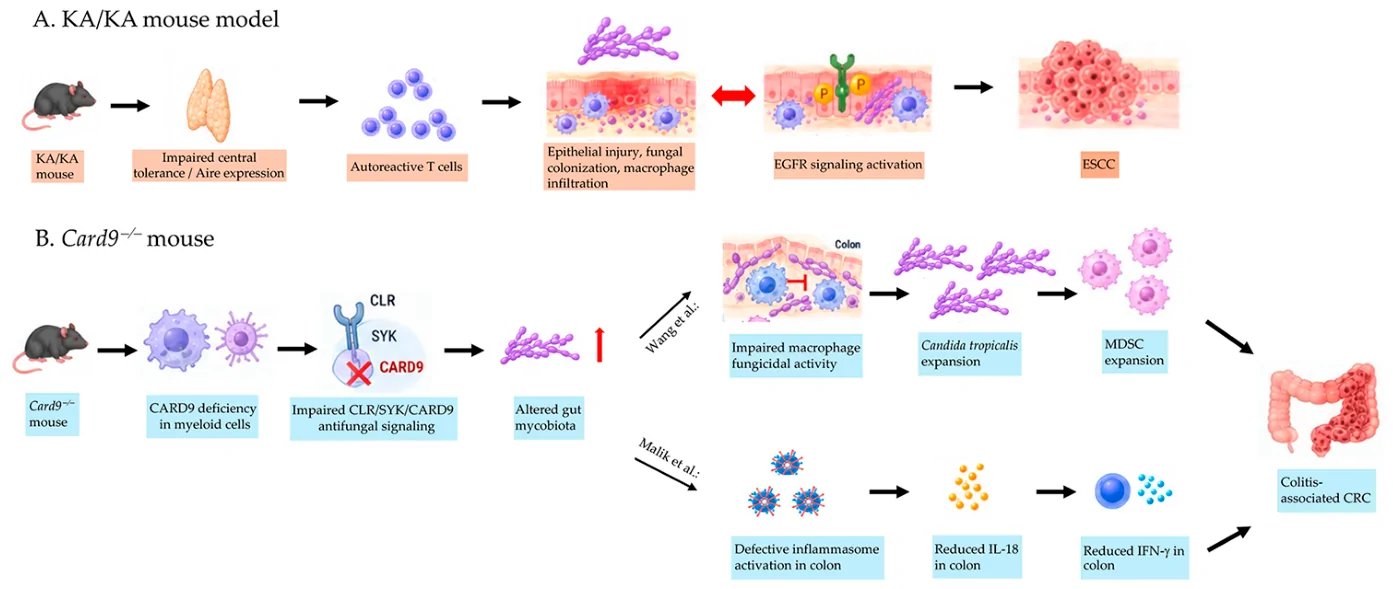
Context-dependent host–fungus interactions in immune dysregulation and GI tumorigenesis. (**A**) KA/KA mouse model. KA/KA mice show impaired central tolerance associated with reduced Aire expression and thymic dysfunction, leading to APECED-like autoimmunity, mucosal inflammation, fungal colonization/infection, epithelial EGFR activation, and ESCC. The red bidirectional arrow denotes the proposed reciprocal relationship between fungal colonization/infection and epithelial EGFR signaling. (**B**) *Card9^−/−^* mouse model. *Card9^−/−^* mice show impaired CLR/SYK/CARD9-dependent antifungal signaling in myeloid cells, resulting in defective macrophage fungicidal activity, increased *Candida* burden or fungal dysbiosis, MDSC expansion, and/or impaired IL-18–IFN-γ antitumor responses, thereby contributing to colitis-associated CRC in a context-dependent manner. The red upward arrow denotes increased fungal burden or dysbiosis.

Together, these findings indicate that KA/KA mice are a useful model for studying how impaired central tolerance, autoimmune inflammation, fungal infection, and epithelial oncogenic signaling cooperate to promote ESCC (Figure 1A). However, the underlying mechanisms require further investigation, including the roles of Th17 responses, neutrophils, macrophages, epithelial barrier dysfunction, and fungal-induced signaling pathways in autoimmune-associated GI tumorigenesis. Table 1 further summarizes KA/KA mice linking fungal infection to ESCC.

## 3. *Card9^−/−^* Mice

The adaptor protein CARD9 is mainly expressed in myeloid cells and is an essential component of host defense, particularly antifungal immunity [41]. Pathogens are detected by host PRRs, including Toll-like receptors (TLRs), CLRs, and Nucleotide-binding oligomerization domain-like receptors (NLRs) [33]. These receptors activate downstream signaling pathways that induce innate immune responses against pathogens. Among these PRRs, CLRs such as Dectin-1 and Dectin-2 serve as important sensors for fungal cell wall components [34]. Dectin-1 primarily recognizes β-glucans, whereas Dectin-2 recognizes a-mannose-containing structures [34]. Upon ligand recognition, these receptors activate SYK-dependent signaling and transmit signals through the CARD9/BCL10/MALT1 complex, leading to activation of downstream pathways, including the NF-κB pathway, and induction of inflammatory signaling cascades [41].

CARD9 deficiency or polymorphism-mediated dysfunction has been linked to immunodeficiency, severe fungal infections, fungal dysbiosis, and autoimmune disorders, including ankylosing spondylitis and IBD [41,52,53,54,55,56]. Although IBD is not classically defined as an autoimmune disease, patients with IBD exhibit systemic overactive immune responses that frequently target intestinal tissues, sharing features with autoimmune inflammation. In addition, patients with IBD have increased risk of CRC [57]. Recent studies using *Card9^−/−^* mice have highlighted important associations among CARD9 deficiency, gut fungal dysbiosis, and AOM-DSS-induced CRC. In these studies, Wang et al. and Malik et al. both implicate CARD9, fungi, colitis, and CRC, but they emphasize different mechanisms [43,44] (Figure 1B).

It has been shown that titers of antifungal antibodies are increased in patients with IBD who have reduced fungal diversity, suggesting fungal dysbiosis and its potential involvement in disease progression [58]. Not surprisingly, Wang et al. found that CRC patients with reduced CARD9 expression in the colon had a higher gut fungal burden, particularly enrichment of *Candida tropicalis* [43] (Figure 1B). This fungal burden positively correlated with the CD11b^+^CD14^−^CD33^+^ MDSC population in peripheral blood. These findings suggest that increased fungal burden may promote colon cancer development through CARD9 deficiency-related macrophage dysfunction and the formation of an immunosuppressive tumor microenvironment shaped by fungus-induced MDSC expansion [43].

Mouse studies have further demonstrated the role of gut fungal burden in colitis-associated colon cancer development [43]. In an AOM-DSS-induced colitis-associated colon cancer model system, Wang et al. showed that CARD9 deficiency markedly increased gut fungal burden in tumor-bearing *Card9^−/−^* mice due to impaired fungicidal activity in CARD9-deficient macrophages. *Candida tropicalis* was identified as the dominant fungal species, whereas the bacterial composition showed no significant changes compared with tumor-bearing wild-type mice. Increased gut fungal burden expanded the MDSC population and resulted in increased tumor burden in *Card9^−/−^* mice. Conversely, reducing fungal burden by antifungal treatment significantly suppressed colon cancer development in AOM-DSS-treated *Card9^−/−^* mice, supporting the idea that increased fungal burden promotes colon cancer development in the setting of CARD9 deficiency.

Notably, inflammatory mediators induced by aberrant gut fungal burden, such as S100A9, IL-6, IL-1β, and Cxcl1, may promote pathogenic MDSC expansion and activation through inflammatory signaling pathways, including TLR-mediated signaling [43].

In contrast, Malik et al. emphasized a protective role for the SYK–CARD9 axis in gut fungal sensing, inflammasome activation, IL-18 maturation, and downstream antitumorigenic IFN-γ responses [44] (Figure 1B). They found that impaired CARD9 signaling reduced protective fungi-induced inflammasome responses and increased susceptibility to colitis and colorectal tumorigenesis. However, restoration of IL-18 signaling or transfer of functional myeloid cells mitigated disease and reduced tumorigenesis, suggesting that fungal recognition through an intact CARD9 pathway can contribute to mucosal protection [44]. Thus, whereas Wang et al. highlighted tumor-promoting consequences of increased fungal burden and MDSC expansion in *Card9^−/−^* mice, Malik et al. emphasized loss of CARD9-dependent protective fungal sensing and impaired IL-18–IFN-γ antitumor immunity.

**Figure 2:**
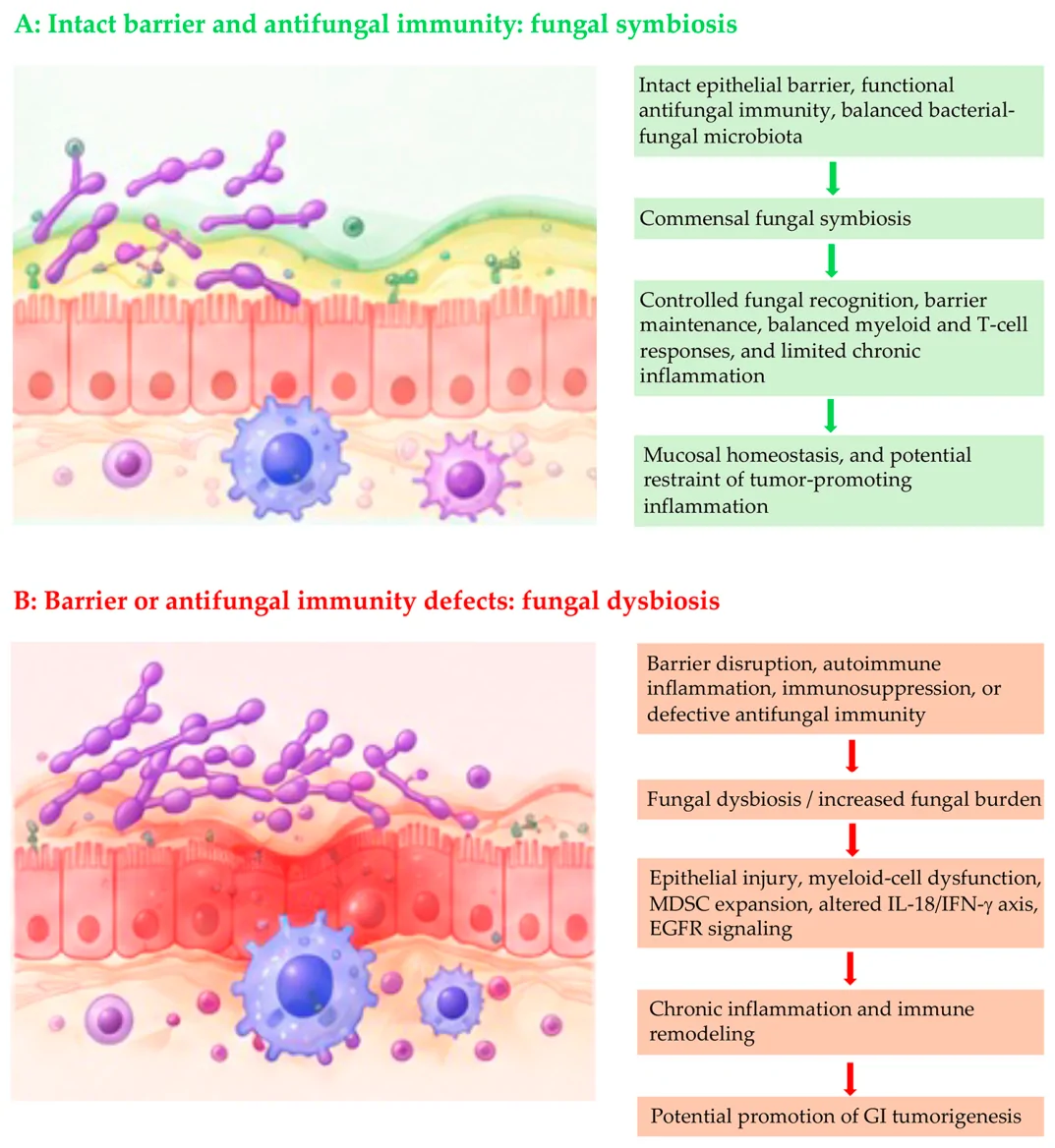
Dual roles of fungi in gastrointestinal tumorigenesis. (**A**) Fungal symbiosis, intact barrier, and antifungal immunity. In immunologically balanced settings, commensal fungi may contribute to mucosal homeostasis by supporting controlled fungal recognition, barrier integrity, antifungal immunity, and limitation of excessive inflammation. (**B**) Fungal dysbiosis, barrier damage, and impaired antifungal immunity. Impaired epithelial barriers and/or antifungal immune pathways may promote fungal dysbiosis or fungal overgrowth, MDSC expansion, and impaired IL-18–IFN-γ responses, generating an immunosuppressive environment that may contribute to GI tumorigenesis.

Together, these studies support a context-dependent model in which gut fungi can exert either pathogenic or protective effects depending on host genotype, antifungal immunity, mycobiota composition, and experimental intervention [59] (Figure 2). However, neither study fully addressed the contributions of neutrophils, Th17 responses, specific fungal metabolites, or bacteria–fungi interactions to CARD9 deficiency-associated fungal dysbiosis and colitis-associated CRC development. Figure 1B and Table 1 summarize distinct mechanisms by which CARD9 may influence gut mycobiota and colitis-associated CRC.

## 4. Discussion

Autoimmune and immune-mediated inflammatory diseases, fungal infections, and cancer are often studied separately. However, recent emerging evidence suggests that these diseases can be mechanistically connected [40,43,44]. Patients with autoimmune diseases exhibit disrupted immune tolerance, chronic inflammation, altered mucosal barriers, as well as frequent exposure to immunosuppressive therapies [60,61,62]. These factors may increase susceptibility to fungal infection or cause fungal dysbiosis. As a result, persistent fungal burden may further amplify inflammation, alter epithelial signaling pathways, reshape local and systemic immunity, and promote an environment favorable for tumor initiation or progression. The KA/KA and *Card9^−/−^* mouse models provide important experimental evidence supporting this concept.

The KA/KA mouse model is particularly valuable because it connects defective central immune tolerance, spontaneous mucosal fungal infection, sustained epithelial EGFR activation, and ESCC. Loss of IKKα kinase activity impairs medullary thymic epithelial cell development and Aire expression, leading to impaired central tolerance, increased autoreactive T cells, and APECED-like autoimmune manifestations [39,40]. Similar to patients with APECED, KA/KA mice develop mucosal fungal infection in the oral cavity and upper GI tract. Importantly, some of these mice spontaneously develop ESCC, and repeated oral inoculation with *C. cladosporioides* markedly increases ESCC incidence. The observation that antifungal treatment reduces fungal burden and prevents ESCC development suggests that fungal colonization is not simply a consequence of tumor formation but may actively contribute to tumorigenesis.

Several mechanisms may explain how fungi promote esophageal cancer in the autoimmune setting. Autoreactive T cells can damage mucosal tissues and generate a chronic inflammatory niche that may permit fungal colonization. Fungal organisms or fungal-derived products may then stimulate epithelial cells, macrophages, and other immune populations, leading to sustained inflammatory signaling and epithelial proliferation. In KA/KA mice, fungal infection has been associated with increased EGFR phosphorylation, whereas EGFR inhibitor treatment reduces fungal colonization in the oral cavity, suggesting that the relationship between fungal colonization and epithelial EGFR signaling is likely bidirectional (Figure 1A). Fungal organisms or fungal-derived products may stimulate epithelial EGFR phosphorylation, thereby promoting epithelial proliferation and survival. Conversely, EGFR activation may alter epithelial barrier function, antimicrobial responses, or the mucosal niche in ways that permit fungal colonization or persistence. In addition, depletion of autoreactive T cells reduces fungal infection, macrophage infiltration, and tumor formation, indicating that autoimmune inflammation and fungal burden reinforce each other during ESCC development. However, the precise contributions of epithelial cells, macrophages, neutrophils, Th17 responses, and fungal metabolites remain to be fully defined.

The *Card9^−/−^* model highlights a different but complementary mechanism. CARD9 is essential for antifungal immunity in myeloid cells. In humans, CARD9 mutations are associated with invasive fungal infections and have also been linked to IBD and CRC susceptibility. In mice, CARD9 deficiency impairs fungal clearance, resulting in increased intestinal fungal burden. In the AOM-DSS model, *Card9^−/−^* mice exhibit impaired macrophage phagosome activity, increased abundance of *Candida tropicalis*, and expansion of MDSCs, and impaired IL-18–IFN-γ antitumor responses, leading to an immunosuppressive tumor microenvironment that may favor tumor development. Nevertheless, in the CARD9 setting, fungi may also exert protective effects depending on host genotype, immune context, fungal species, and mycobiome structure. Similar to KA/KA mice, antifungal treatment also suppresses colon cancer development in these mice, supporting a causal role for fungal burden or fungal dysbiosis in promoting CRC under conditions of impaired antifungal immunity.

The KA/KA and *Card9^−/−^* models therefore illustrate two related but distinct routes by which fungi may contribute to GI cancer. In KA/KA mice, defective central tolerance produces autoimmune inflammation that creates a permissive environment for fungal infection and esophageal tumorigenesis. In *Card9^−/−^* mice, defective innate antifungal immunity leads to intestinal fungal dysbiosis, and immune suppression, which may promote CRC development. Despite these differences, both models support a shared principle: when immune homeostasis is disrupted, fungi can become active participants in cancer-promoting inflammation and immune remodeling.

These findings have potential clinical relevance but should currently be viewed as exploratory. In selected high-risk populations, such as patients with APECED, chronic mucosal candidiasis, STAT1 gain-of-function disease [63], or longstanding IBD, future studies could evaluate whether mucosal fungal burden, antifungal antibody profiles, or mycobiome features correlate with cancer risk, inflammatory activity, or treatment response. However, no validated fungal assay currently exists for GI cancer risk stratification, and prospective clinical data are lacking. Similarly, antifungal intervention should not be viewed as a cancer-preventive strategy outside established indications for treating fungal infection. Because fungal communities may include pathogenic, neutral, or protective organisms, broad antifungal prophylaxis could have unintended consequences.

Several important questions remain unresolved. First, it is unclear which fungal species are most relevant to autoimmune or immune-mediated inflammation-associated GI cancer in humans. Although *C. cladosporioides* and *C. tropicalis* have been implicated in mouse models, human studies are needed to determine whether these fungi have similar tumor-promoting or pro-inflammatory roles in patients. Interestingly, a recent study reported that *C. cladosporioides* was enriched in human HNSCC samples [49], although whether these patients had autoimmune or immune-mediated inflammatory manifestations remains unknown. It will also be important to distinguish protective fungal commensalism from pathogenic fungal dysbiosis. In healthy mucosal tissues, commensal fungi may contribute to immune education, stimulate antifungal antibody responses, and help maintain balanced mucosal immunity. However, changes in fungal species composition, fungal burden, and epithelial barrier integrity may allow commensal fungi to become pathogenic. For example, broad antifungal treatment may remove potentially protective fungal signals and alter bacterial–fungal ecological networks. Second, the fungal-derived molecules responsible for epithelial activation, inflammatory cell recruitment, or immune suppression remain poorly characterized. Third, the timing of fungal colonization relative to autoimmune or immune-mediated inflammation and tumor initiation needs to be defined. Longitudinal studies in patients and controlled experiments using germ-free or defined fungal-colonized mouse models will be particularly useful. Finally, interactions among fungi, bacteria, viruses, diet, host genetics, and immunosuppressive therapies should be investigated, because GI cancer likely arises from complex microbial and immune networks rather than from a single organism alone.

## 5. Conclusions

The KA/KA and *Card9^−/−^* mouse models provide useful tools for investigating how fungi may connect immune dysregulation with GI tumorigenesis in a context-dependent manner. The KA/KA model highlights defective central tolerance, autoreactive T-cell-mediated mucosal inflammation, fungal colonization/infection, epithelial EGFR activation, and ESCC. By contrast, the *Card9^−/−^* model emphasizes impaired innate antifungal immunity, altered gut mycobiota composition, myeloid-cell dysfunction, MDSC expansion, and impaired IL-18–IFN-γ antitumor responses in colitis-associated CRC. Together, these models support the hypothesis that fungi may contribute to cancer-promoting inflammation and immune remodeling in selected immune-dysregulated settings. However, these findings should not be interpreted as broadly representative of all autoimmune or immune-mediated inflammatory diseases. Further mechanistic and human studies are needed.

## Figures and Tables

**Table 1:** Summary of KA/KA and *Card9^−/−^* mouse models linking host–fungus interactions to GI cancer.

Model/ Disease	Genetic Defect	Disease Context	Fungal Finding	Intervention	Effect on Inflammation/ Tumorigenesis	Demonstrated Mechanism	Proposed/ Remaining Questions	Evidence Level
KA/KA mice [39,40]	Kinase-dead IKKα	APECED-like autoimmune phenotype	Oral/ esophageal fungi, including *Cladosporium* spp.	Repeated *C.* *cladosporioides* inoculation	Increased ESCC incidence	Autoreactive T cells, inflammation, macrophage infiltration, epithelial EGFR activation associated with tumorigenesis	Th17/ neutrophil roles, epithelial barrier mechanisms	Experimental mouse model
KA/KA mice	Kinase-dead IKKα	Autoimmune- associated ESCC	Increased Fungal burden	Antifungal treatment	Reduced tumor burden and prevented/ Reduced ESCC development	Fungal burden contributes to tumor- promoting environment	Specific Fungal Species and causal pathways require further study	Experimental mouse model
*Card9^−/−^* mice, Wang et al. [43]	CARD9 deficiency	AOM-DSS colitis- associated CRC	Increased fungal burden, *Candida* *tropicalis* enrichment	Antifungal treatment	Reduced CRC burden	Impaired macrophage fungicidal activity; MDSC expansion	Species specificity, bacterial- fungal interactions, timing of intervention	Experimental mouse model plus human association
*Card9^−/−^* mice, Malik et al. [44]	CARD9 deficiency	Colitis and CAC	Impaired fungal sensing through SYK-CARD9 pathway	IL-18 restoration or functional myeloid cell transfer	Reduced Disease susceptibility	CARD9- dependent inflammasome activation IL-18 maturation, IFN-γ responses	How protective fungal sensing balances pathogenic fungal overgrowth	Experimental mouse model

## Data Availability

No new data were created or analyzed in this study. Data sharing is not applicable to this article. All data discussed in this manuscript are available in the cited literature.
